# Cellulose Acetate Butyrate-Based In Situ Gel Comprising Doxycycline Hyclate and Metronidazole

**DOI:** 10.3390/polym16243477

**Published:** 2024-12-13

**Authors:** Ei Mon Khaing, Nutdanai Lertsuphotvanit, Warakon Thammasut, Catleya Rojviriya, Siraprapa Chansatidkosol, Supanut Phattarateera, Wiwat Pichayakorn, Thawatchai Phaechamud

**Affiliations:** 1 Program of Pharmaceutical Engineering, Department of Industrial Pharmacy, Faculty of Pharmacy, Silpakorn University, Nakhon Pathom 73000, Thailand; khaing_e@su.ac.th (E.M.K.); thammasut_w@silpakorn.edu (W.T.); 2 Faculty of Pharmaceutical Sciences, Burapha University, Chonburi 20131, Thailand; nutdanai.le@go.buu.ac.th; 3 Synchrotron Light Research Institute, Nakhon Ratchasima 30000, Thailand; catleya@slri.or.th; 4 Faculty of Science and Technology, Rajamangala University of Technology Krungthep, Bangkok 10120, Thailand; siraprapa.ch@mail.rmutk.ac.th; 5 Plastic Technology Research Team, Advanced Polymer Research Group, National Metal and Materials Technology Center (MTEC), Pathum Thani 12120, Thailand; supanut.pha@mtec.or.th; 6 Department of Pharmaceutical Technology, Faculty of Pharmaceutical Sciences, Prince of Songkla University, Songkhla 90110, Thailand; wiwat.p@psu.ac.th; 7 Department of Industrial Pharmacy, Faculty of Pharmacy, Silpakorn University, Nakhon Pathom 73000, Thailand

**Keywords:** cellulose acetate butyrate, in situ gel, metronidazole, doxycycline hyclate, combination

## Abstract

Cellulose acetate butyrate is a biodegradable cellulose ester bioplastic produced from plentiful natural plant-based resources. Solvent-exchange-induced in situ gels are particularly promising for periodontitis therapy, as this dosage form allows for the direct delivery of high concentrations of antimicrobial agents to the localized periodontal pocket. This study developed an in situ gel for periodontitis treatment, incorporating a combination of metronidazole and doxycycline hyclate, with cellulose acetate butyrate serving as the matrix-forming agent. Consequently, assessments were conducted on the physicochemical properties, gel formation, drug permeation, drug release, morphological topography, and antimicrobial activities of the formulation. The formulation demonstrated an increased slope characteristic of Newtonian flow at higher bioplastic concentrations. The adequate polymer concentration facilitated swift phase inversion, resulting in robust, solid-like matrices. The mechanical characteristics of the transformed in situ gel typically exhibit an upward trend as the polymer concentration increased. The utilization of sodium fluorescein and Nile red as fluorescent probes effectively tracked the interfacial solvent–aqueous movement during the phase inversion of in situ gels, confirming that the cellulose acetate butyrate matrix delayed the solvent exchange process. The initial burst release of metronidazole and doxycycline hyclate was minimized, achieving a sustained release profile over 7 days in in situ gels containing 25% and 40% cellulose acetate butyrate, primarily governed by a diffusion-controlled release mechanism. Metronidazole showed higher permeation through the porcine buccal membrane, while doxycycline hyclate exhibited greater tissue accumulation, both influenced by polymer concentration. The more highly concentrated polymeric in situ gel formed a uniformly porous structure. Metronidazole and doxycycline hyclate-loaded in situ gels showed synergistic antibacterial effects against *S. aureus* and *P. gingivalis*. Over time, the more highly concentrated polymeric in situ gel showed superior retention of antibacterial efficacy due to its denser cellulose acetate butyrate matrix, which modulated drug release and enhanced synergistic effects, making it a promising injectable treatment for periodontitis, particularly against *P. gingivalis*.

## 1. Introduction

The primary pathogens that invade the superficial gingival tissues and accumulate in deep crevicular pockets are predominantly anaerobic bacteria, particularly *Porphyromonas gingivalis*, which is a key cause of periodontitis [1]. Some pathobionts facilitate the formation of dysbiotic microbial communities, which can undermine the host immune system, leading to inflammation and tissue damage. *Gram*-negative, obligate anaerobic bacteria such as *P. gingivalis* have been strongly associated with periodontitis [2]. The presence of *P. gingivalis* is often linked to periodontal deterioration due to its numerous virulence factors that effectively stimulate host immune responses. Its presence elevates the risk of developing periodontal disease, making it a notable contributor to the initiation of the progression of disease [1]. Other bacterial species linked to periodontitis include *Prevotella intermedia*, *Fusobacterium nucleatum*, *Selenomonas*, and *Parvimonas micra* [3]. These *Gram*-negative anaerobes are notoriously difficult to eliminate due to their robust cell walls, particularly when they accumulate in deep crevicular pockets. Beneath their capsule, *Gram*-negative bacteria possess an outer membrane that shields them from certain antibiotics, and when this membrane is disrupted, it releases endotoxins [4]. These endotoxins exacerbate the symptoms during infections caused by *Gram*-negative bacteria. Furthermore, the growing issue of antibiotic resistance among anaerobes has increasingly complicated the selection of effective therapeutic agents.

**Figure 1:**
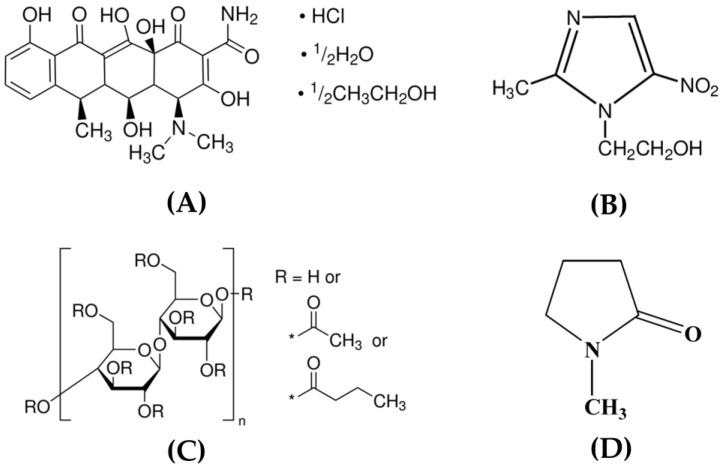
Chemical structures of doxycycline hyclate (**A**), metronidazole (**B**), cellulose acetate butyrate (**C**), and *N*-methyl pyrrolidone (NMP) (**D**).

To minimize side effects and adverse drug reactions associated with systemic antibiotic administration, localized drug delivery targeting the crevicular pocket in periodontitis offers a promising alternative. Various antiseptic and antibiotic agents have been incorporated into different delivery systems, such as chips, films, strips, gels, and microparticles. Among these, doxycycline, known for its antibacterial and anti-inflammatory properties, also supports wound healing and influences alveolar bone metabolism [5,6,7]. As a broad-spectrum antibiotic from the tetracycline class, doxycycline is widely used. It can enhance the regenerative potential of periodontal ligament mesenchymal stem cells, modulated by IL-17, particularly in cell migration [5]. Doxycycline acts as a bacteriostatic agent by inhibiting protein synthesis. It achieves this by reversibly binding to the 30S subunit of the ribosome in susceptible organisms, thereby preventing the attachment of aminoacyl transfer RNA [6]. The doxycycline hyclate (Figure 1A) used in this study is one of the active compounds. It appears as a yellow-green crystalline solid with a molecular weight of 526.97 g per mole and a melting point ranging from 206 to 209 °C. This antibiotic drug has been incorporated into local drug delivery systems for periodontitis therapy [7,8]. Metronidazole (Figure 1B), belonging to the nitroimidazole class, serves as a primary anti-anaerobic agent. Its antimicrobial action is based on the reduction of its nitro group (-NO_2_) to an amino group (-NH_2_) through the activity of ferredoxin in anaerobic microorganisms. This reduction generates a product that damages microbial DNA, ultimately leading to cell death [9]. Metronidazole-loaded porous matrices composed of gelatin and cellulose derivatives have been reported for localized periodontitis therapy [10], and metronidazole-containing thermosensitive poloxamer gels have been developed for similar treatments [11]. Additionally, doxycycline hyclate–metronidazole combinations have demonstrated synergistic antimicrobial activity against *P. gingivalis* using the checkerboard method [12]. Solid lipid microparticles encapsulating doxycycline hydrochloride, combined with a metronidazole-loaded poloxamer gel, were developed for direct injection into periodontal pockets as an adjunct to scaling and root planing in the treatment of periodontal diseases [13]. Therefore, the incorporation of these two active compounds into a drug delivery system presents a promising approach for periodontitis therapy.

Currently, in situ gels formed through phase inversion and solvent exchange are used to target antibiotic drugs directly toward periodontal pockets as a treatment for periodontitis. One such commercially available product, Atridox^®^, contains doxycycline hyclate as its active antibacterial agent and is administered by a dentist via injection to patients with periodontitis [7,8]. This formulation is a drug-dissolved poly(DL-lactide) (PLA) solution or suspension that, once injected into the periodontal pocket, solidifies into a polymer matrix that controls the drug release over an extended duration [7]. For optimal therapeutic outcomes, the in situ gel must exhibit appropriate viscosity, efficient gelation, ease of injection, and prolonged drug release, while effectively fighting microbial infections caused by relevant pathogens. The fluid properties of solvent-exchange-induced in situ gels in their solution state are influenced by the choice of polymer and solvent, which highlights the significance of selecting the appropriate polymer type, polymer concentration, and solvent when developing this dosage form [7,14].

Cellulose acetate butyrate (Figure 1C) is a cellulosic bioplastic polymer derived from the esterification of cellulose with acetic and butyric acids [15]. A higher content of butyrate enhances the polymer’s hydrophobicity, flexibility, and solubility in organic solvents [16]. Cellulose acetate butyrate demonstrates strong thermal stability, attributed to its cellulose backbone, and increasing the butyrate content lowers the glass transition temperature (Tg), thus improving the polymer’s flexibility. Its decomposition temperature typically exceeds 200 °C [15,16], making it ideal for applications that require heat resistance. Additionally, cellulose acetate butyrate’s biocompatibility makes it suitable for use in controlled-release drug delivery systems. Its hydrophobic characteristics allow it to function as a matrix former in sustained-release formulations, where it regulates drug release by controlling both diffusion and erosion processes. Cellulose acetate butyrate membranes have demonstrated superior stability when compared to cellulose acetate membranes in osmotic drug delivery systems [16]. Cellulose acetate butyrate is water-insoluble and non-ionizable, making its pseudolatex suitable for pH-independent coatings in controlled drug delivery applications [17]. Due to its insolubility in water and bioplastic properties, cellulose acetate butyrate possesses desirable characteristics for use as a matrix-forming agent in solvent-exchange-induced in situ gels. Our research group found that cellulose acetate butyrate was able to dissolve in *N*-methyl-2-pyrrolidone (NMP) (Figure 1D); consequently, this finding could open up potential applications, particularly in the development of in situ gel systems. NMP is a polar, aprotic solvent commonly used in pharmaceutical formulations, including in situ gel systems, owing to its good solubilizing power [14]. This solvent is ideal for developing injectable or implantable in situ gels, where the solvent exchange induces polymer precipitation upon contact with aqueous environments, leading to gel formation at the site of administration [18]. The fabrication of benzydamine HCl or moxifloxacin HCl-loaded cellulose acetate butyrate in situ gels, each containing a single drug, was achieved using straightforward, non-sophisticated techniques [18,19]. These gels offer sustained drug release along with potent antimicrobial activity. However, there have been no previous reports on the development or in-depth investigation of solvent-exchange-based in situ gels made from this cellulosic bioplastic polymer, cellulose acetate butyrate, loaded with doxycycline hyclate–metronidazole combinations for the treatment of periodontitis.

This investigation aimed to develop an in situ gel formulation utilizing phase inversion and solvent exchange, with cellulose acetate butyrate serving as the matrix-forming agent, for the treatment of periodontitis by incorporating a combination of doxycycline hyclate and metronidazole as the active compounds. To achieve this, the matrix-forming behavior of cellulose acetate butyrate at various concentrations was examined. The effects of this cellulosic bioplastic polymer concentration were subsequently evaluated and discussed in relation to its physicochemical properties, matrix formation, dual-drug release, and permeation, as well as its antimicrobial activities.

## 2. Materials and Methods

### 2.1. Materials

Doxycycline hyclate (Batch No. 20071121, Huashu Pharmaceutical Corporation, Shijiazhuang, China) was provided by Bangkok Lab and Cosmetic Public Company Ltd., Rachaburi, Thailand. Metronidazole was generously supplied by T.MAN Pharma Ltd., Bangkok, Thailand. Cellulose acetate butyrate (CAB 551-0.01), with an acetyl content of 2% w/w, butyryl content of 52% w/w, hydroxyl content of 2% w/w, and M.W. of approximately 50 to 70 kDa (lot No. RP-000936), was sourced from Eastman, Katzbergstrasse, Langenfeld, Germany, and served as the gel-forming agent. NMP (Lot No. 144560-118, QReC, Auckland, New Zealand) was used as the solvent for dissolving drugs and cellulose acetate butyrate. Potassium dihydrogen orthophosphate (lot no. E23W60) and sodium hydroxide (lot no. AF310204) from Ajax Finechem, New South Wales, Australia, were used to prepare phosphate-buffered saline (PBS). Agarose (Lot No. H7014714, Vivantis, Selangor Darul Ehsan, Malaysia) was employed to investigate gel formation and interfacial phase transformation. Acetonitrile (HPLC grade), used for HPLC analysis, was purchased from RCI Labscan, Bangkok, Thailand. Sodium fluorescein (lot no. SHBL6563) and Nile red (lot no. BCBP8959V) (Sigma-Aldrich, Inc., St. Louis, MO, USA) were used for studying interfacial interactions.

For antimicrobial testing, sheep blood agar (SBA) (Ministry of Public Health, Nonthaburi, Thailand) was employed alongside tryptic soy agar (TSA) and tryptic soy broth (TSB) (Difco™, Detroit, MI, USA). For antifungal testing, Sabouraud dextrose agar (SDA) and Sabouraud dextrose broth (SDB) (Difco™, Detroit, MI, USA) were utilized. The strains Staphylococcus aureus ATCC 6538 and Candida albicans ATCC 10,231 were obtained from the Department of Medical Sciences, Ministry of Public Health, Nonthaburi, Thailand. Additionally, Porphyromonas gingivalis ATCC 33,277, sourced from Microbiologics Inc., St. Cloud, MN, USA, was provided by Thai Can Biotech Co., Ltd., Bangkok, Thailand, for use as a test microorganism.

### 2.2. Preparation of Metronidazole and Doxycycline Hyclate-Dissolved Cellulose Acetate Butyrate In Situ Gels

A 2% w/w metronidazole solution and a 2% w/w doxycycline hyclate solution in NMP were prepared as control groups, dissolved, and stirred in glass bottles. Additionally, formulations containing 2% w/w metronidazole and 2% w/w doxycycline hyclate in varying concentrations of cellulose acetate butyrate (10, 15, 20, 25, 30, 35, 40, and 45% w/w) dissolved in NMP were prepared. The weight of each component was calculated as a percentage of the total formulation weight to ensure accurate proportions. The mixtures were stirred at 25 °C using a magnetic stirrer (Benchmark H3770-HS-E Digital Hotplate Stirrer, Benchmark Scientific Inc., Sayreville, NJ, USA) in glass bottles until clear solutions were achieved. Similarly, 2% w/w metronidazole in 40% w/w cellulose acetate butyrate dissolved in NMP and 2% w/w doxycycline hyclate in 40% w/w cellulose acetate butyrate dissolved in NMP were prepared through continuous mixing in glass bottles until clear solutions were obtained. Control formulations containing 40% w/w cellulose acetate butyrate in NMP were also prepared. The components of all formulations are presented in Table 1.

### 2.3. Evaluations

#### 2.3.1. Physical Appearance and Measurements of Viscosity and Rheology

Following the preparation of all formulations listed in Table 1, their physical appearance was assessed, focusing on factors such as clarity, color, and the presence of precipitates. Measurements of viscosity and rheological behavior were conducted using a cone-plate viscometer (RM 100 CP2000 plus, Lamy Rheology Instruments, Champagne-au-Mont-d’Or, France) at 25 °C. The measurements were conducted at 15 s intervals using a cone with a 10 mm diameter and a 70 mm bottom plate. To enable a consistent comparison of viscosity among different formulations, the shear rate was maintained at 120 rpm, and the viscosity values were recorded under these conditions. Each measurement was performed in triplicate.

#### 2.3.2. Determination of Contact Angle

The sessile drop method, using an optical tensiometer, is employed to determine the contact angle of a liquid on a surface, providing valuable insights into the surface’s wetting properties in relation to the liquid droplet. In this study, the sessile drop method was applied to assess the spreadability of formulation droplets and to investigate the role of phase inversion in droplet spreadability on both dry and wet surfaces. This approach allowed for a better understanding of how the formulations behave under different surface conditions. Contact angle measurements were performed using a drop shape analyzer (FTA 1000, First Ten Angstroms, Newark, CA, USA) via the sessile drop method. The tests were conducted on both glass slides and agarose gel surfaces, with a droplet pump-out rate of 1.9 µL/s through a 14-gauge needle, as previously mentioned [20]. This allowed for consistent evaluation of droplet behavior across different surface types. The contact angle was recorded 5 s after droplet placement, and each measurement was performed in triplicate.

#### 2.3.3. Injectability

The injectability of all formulated preparations was evaluated using a texture analyzer in compression mode (TA.XT Plus, Stable Micro Systems, Godalming, UK). Each formulation was added into a 1 mL plastic syringe equipped with a 21-gauge needle with a length of 25 mm, which was secured to a stainless-steel stand for the analysis. The diameter of the piston of this plastic syringe was 4.7 mm. The upper probe of the instrument was driven downward at a constant speed of 1.0 mm·s^−1^, applying a force of 0.1 N to the base of the syringe barrel as previously reported [14,20]. The area under the resulting force–time curve was recorded to determine the work of the injection (N·mm) (n = 3).

#### 2.3.4. Test of Mechanical Property

The study assessed the mechanical property of an in situ gel after undergoing solvent exchange within a 7 mm diameter hollow agarose gel containing PBS (pH 6.8) for 3 days as previously described [20,21]. The gel’s mechanical behavior was analyzed using a texture analyzer (TA.XT plus, Stable Micro Systems, Surrey, UK). A stainless steel spherical probe, 5 mm in diameter, was gradually pressed into the obtained matrix at a constant speed of 0.5 mm/s. The relationship between the applied force and probe displacement over time was recorded, and the maximum penetration force was measured (n = 3).

#### 2.3.5. Test of Gel Formation in Aqueous Environments

To evaluate the ability for phase inversion due to solvent exchange, which induces polymer phase separation, the transition from a solution state to a gel or solid matrix-like state was assessed. A 1 mL aliquot of the prepared formulation was injected through an 18-gauge stainless steel needle into 5 mL of PBS (pH 6.8) in a glass test tube. The morphological changes were photographed at various time intervals (1, 5, and 10 min) to capture the phase transition.

#### 2.3.6. Observation of Microscopic Interfacial Change

The phase interaction at the interface between the formulation and the aqueous fluid of agarose was studied to elucidate phase transformation using a method adopted from previous research [19]. A 0.6% agarose solution was added onto a glass slide, forming a 2 mm thick gel layer. This layered gel was carefully cut into a smooth, straight edge positioned 2 cm from one side of the glass slide. Next, this prepared agarose gel layer was placed adjacent to a 20 µL dosage form near the incision edge. The phase changes resulting from phase transition at the interface, triggered by exposure to PBS (pH 6.8) within the agarose gel, were observed and recorded using an inverted stereomicroscope (Nikon Eclipse TE2000S, Nikon, Kawasaki, Japan) at different time intervals (1, 5, and 10 min).

To monitor the diffusion of aqueous and NMP phases during phase transformation, fluorescent tracking was employed using a hydrophilic compound (sodium fluorescein) and a hydrophobic compound (Nile red), following a technique adapted from previous research [18]. Four different conditions were investigated:

(a) The 0.2 μg/mL sodium fluorescein-loaded agarose gel with the formulation.
(b) The agarose gel with the 0.2 μg/mL sodium fluorescein-loaded formulation.
(c) The 0.2 μg/mL sodium fluorescein-loaded agarose gel with the 0.4 μg/mL Nile red-loaded formulation.
(d) The 0.4 μg/mL Nile red-loaded formulation with the agarose gel.

Fluorescent color changes at the interface were observed using an inverted fluorescence microscope (Nikon Eclipse TE2000S, Nikon, Kawasaki, Japan) at 40× magnification. A blue (B2A) filter, excited at 450–490 nm, was used to visualize the green fluorescence of sodium fluorescein, while a green (G2A) filter, excited at 510–560 nm, was used to track the red fluorescence of Nile red. Images were captured at 1, 3, 5, and 10 min to monitor changes in fluorescence at the interface.

#### 2.3.7. The Drug Content and In Vitro Dual-Drug Release Test

The content of doxycycline hyclate and metronidazole in the formulated MtN, DhN, MtDh25C, and MtDh40C samples were determined using high-performance liquid chromatography (HPLC) analysis carried out on an Agilent 1260 system, which included a photodiode array detector (Agilent Technologies Singapore Pte Ltd., Singapore). Separation was conducted using an ACE C18 column (dimensions: 4.6 × 250 mm, particle size: 5 µm) supplied by VWR International, PA, USA (n = 6). The analysis followed a standard curve. The mobile phase consisted of a 7:3 mixture of 0.1% phosphoric acid in water and acetonitrile, applied in isocratic elution at a flow rate of 0.5 mL/min. An injection volume of 20 µL was used, with detection wavelengths set at 320 nm for metronidazole and 347 nm for doxycycline hyclate. The total analysis time for each run was 20 min. The developed method was validated for specificity, linearity and range, limit of detection (LOD), and limit of quantification (LOQ).

A cylindrical porcelain cup with a diameter of 1 cm and a height of 1.2 cm, containing 0.4 g of the formulation, was submerged in 50 mL of PBS pH 6.8 at 37 °C and stirred at 50 rpm to simulate drug release from a crevicular pocket [14,18]. At predetermined intervals, 5 mL samples of the release medium were withdrawn and replaced with an equal volume of fresh PBS. The concentrations of the dual drugs released were measured using the previously described HPLC method. Each experiment was performed in triplicate. Prior to HPLC analysis, the collected samples were filtered through a 0.22 µm nylon membrane to ensure accurate drug quantification. Subsequently, the drug release mechanisms were analyzed by curve fitting the obtained dissolution data to various mathematical release models, including zero-order, first-order, Higuchi’s, Korsmeyer–Peppas, and Peppas–Sahlin models. A higher degree of fit was indicated by elevated values of the calculated coefficient of determination (R^2^), along with lower values of the Akaike Information Criterion (AIC) and higher values of the Model Selection Criterion (MSC). This analysis process was undertaken using the DD-Solver^®^ software version 1.0, a Microsoft Excel add-in (Redmond, WA, USA) developed in Visual Basic for Applications [22].

#### 2.3.8. Characterization of Transformed In Situ Gel

##### Topography Observation Using Scanning Electron Microscope (SEM)

SEM analysis was conducted to investigate the detailed surface and cross-sectional morphology of the prepared formulations after solvent removal, enabling high-resolution visualization of the topographical features. After the matrix formation was confirmed following a 7-day drug release period, the remaining samples were thoroughly washed multiple times with distilled water to eliminate residual impurities. The samples were then carefully dried in a desiccator for one week. Once dried, the remnants were coated with a thin layer of gold using a conventional sputtering device (BIO-RAD SEM Coating Unit PS3, BIO-RAD Laboratories Ltd., Hercules, CA, USA). The coated remnants were examined using a field-emission SEM (TESCAN MIRA3, Brno-Kohoutovice, Czech Republic) at magnifications of 30×, 1000×, and 10,000×, with an accelerating voltage of 15 kV.

##### Topography Observation via Synchrotron Radiation X-Ray Tomographic Microscopy (XTM)

Synchrotron Radiation X-ray Tomographic Microscopy (XTM) was utilized to observe the internal and surface topography of the samples in three dimensions. This technique provides high-resolution, non-destructive imaging, facilitating detailed analysis of the microstructure, including phase distribution, pore morphology, and material density variations. The collected remnants dried and prepared for SEM analysis were further examined using X-ray tomography (XTM) at the Synchrotron Light Research Institute (SLRI) beamline in Nakhon Ratchasima, Thailand. Synchrotron radiation X-rays were produced by a 2.2-T multipole wiggler at the Siam Photon Source, operating at 1.2 GV with a beam current of 150 mA. The experiments utilized a filtered polychromatic X-ray beam with an average energy of 12.5 keV and a source-to-sample distance of 34 m. Sample projections were captured using a detection system comprising a 200 µm thick scintillator (YAG, Crytur, Turnov, Czech Republic), a 2× objective lens-coupled X-ray microscope (Optique Peter, Lentilly, France), and an sCMOS camera (pco.edge 5.5, 2560 × 2160 pixels, 16-bit resolution) (Optique Peter, Lentilly, France).

Tomographic scans were performed with an isotropic voxel size of 3.61 µm. After data collection, the X-ray projections were normalized using a flat-field correction algorithm and reconstructed into tomographic slices with the Octopus reconstruction software version 8.9.1 (TESCAN, Gent, Belgium). A 3D representation of the tomographic volumes of the test sample and segmentation analysis was computed using Drishti software version 2.6.4 (National Computational Infrastructure’s VizLab, The Australian National University, Canberra, ACT, Australia). The test specimens were analyzed for their porosity in three dimensions via segmentation analysis using Octopus Analysis (TESCAN, Gent, Belgium) [18,23]. This thorough methodology enabled a detailed investigation of the internal structure and morphology of the dried remnants of cellulose acetate butyrate-based in situ gels.

#### 2.3.9. Ex Vitro Permeation Study

To assess the penetration of these antibiotic drugs and their potential to inhibit invasive pathogens within periodontal pocket tissues, an ex vitro permeation study was conducted. However, using actual periodontal pocket tissue as the membrane is impractical due to its small size. Therefore, this study adapted fresh buccal mucosa as the test membrane in a Franz diffusion cell for drug permeation analysis, as previously reported [20]. Fresh porcine bulge tissues were sourced from a local slaughterhouse in Nakhon Pathom province, Thailand. Their subcutaneous fatty layer and connective tissues were carefully removed from the protruding area of the porcine cheek tissue. The attained cleaned buccal mucosa membrane was subsequently rinsed with PBS at pH 7.4 and then stored at −4 °C and moved to a 4 °C environment one day prior to the experiments. The stored membrane was equilibrated in PBS at pH 7.4 and 25 °C for two hours before testing. A circular section of the membrane (3 × 3 cm^2^) was mounted in a Franz diffusion cell, with the external buccal side facing the donor compartment. The receiver compartment (13 mL) was filled with PBS (pH 6.8) for the permeation study, maintained at 37 ± 1 °C, and continuously stirred using a magnetic stirrer.

At specific time intervals over 24 h, 2 mL of the solution from the receiver compartment was collected and replaced with an equal volume of fresh medium. The drug permeation through the buccal membrane into the receptor fluid, as well as the remaining amounts of doxycycline hyclate and metronidazole in the donor compartment, buccal membrane, and receptor fluid, were quantified using the previously described HPLC techniques (n = 3). The cumulative permeation of both active compounds through the buccal membrane per unit area was determined based on each compound’s concentration in the receiving medium, relative to the membrane’s contact surface area, and plotted over time. The flux was calculated as the slope of the plot’s linear section, and the lag time was derived from the linear equation of that same plot. Additionally, the remaining contents of both drugs in the donor compartment, along with their accumulation amount within the buccal membrane, were quantified using HPLC and an extraction technique as previously reported (n = 3) [20].

#### 2.3.10. Antimicrobial Activity Test

The bioactivity of the prepared formulations was evaluated by assessing their antimicrobial properties against *S. aureus* ATCC 6538, *C. albicans* 10231, and *P. gingivalis* ATCC 33,277 using an agar diffusion assay (cylinder plate method), following a previously reported technique [7]. In brief, *S. aureus* ATCC 6538 inoculated in TSB, adjusted to a turbidity equivalent to the 0.5 McFarland standard, was spread on TSA, while *C. albicans* 10231 inoculated in SDB was spread on SDA. For *P. gingivalis*, the inoculum, adjusted to a similar turbidity, was spread on SBA. A 100 µL aliquot of each prepared formulation was added to the cylinder cap. The plates for *S. aureus* ATCC 6538 and *C. albicans* 10231 were incubated in a standard incubator (Thermo Scientific Precision Compact Incubators, Thermo Scientific, Cincinnati, OH, USA). *P. gingivalis* was incubated anaerobically using a Forma Anaerobic System (Thermo Scientific, Cincinnati, OH, USA). MtN and DhN served as positive controls, while 40C and NMP were used as negative controls. After 24 h of incubation at 37 °C, the inhibition zone diameters for each microorganism were measured with a standard ruler (n = 3). The sustainability of antimicrobial activity in the in situ gel formulations was evaluated by repeatedly transferring the previous formulation-loaded cup into freshly inoculated agar media. This process was repeated to observe any changes in the diameter of the inhibition zone against *S. aureus*, *C. albicans*, and *P. gingivalis* over intervals on days 1, 5, 9, and 15 (n = 3).

### 2.4. Statistical Analysis

The data were expressed as the mean ± standard deviation (S.D.). Statistical significance was assessed using a one-way analysis of variance (ANOVA), followed by the least significant difference (LSD) post hoc test, performed with SPSS software for Windows (version 11.5). A *p*-value of less than 0.05 was considered statistically significant, indicating a meaningful difference in the results.

## 3. Results and Discussion

### 3.1. Physical Appearance, Viscosity and Injectability

**Figure 2:**
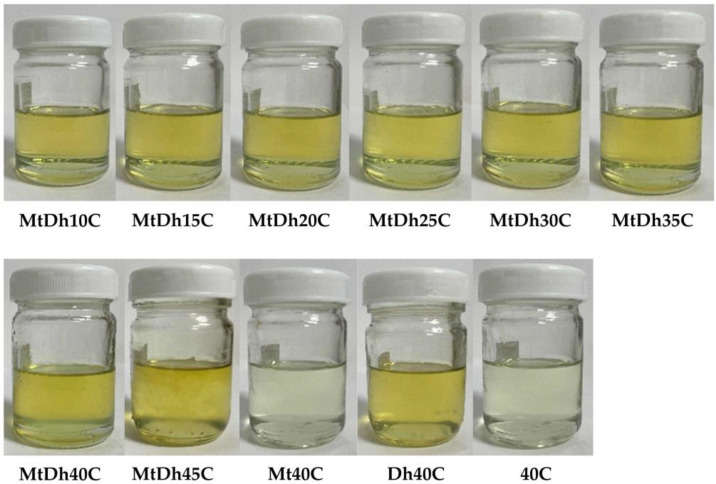
Physical appearance of metronidazole combined with doxycycline hyclate-dissolved cellulose acetate butyrate-based in situ gel and control formulations.

All prepared formulations containing metronidazole and doxycycline hyclate appeared as yellowish transparent solutions, resembling the single drug-loaded solution (Dh40C) due to the inherent color of doxycycline hyclate (Figure 2). In contrast, formulations without doxycycline hyclate produced a clear, colorless solution. When cellulose acetate butyrate was incorporated at levels above 45%, it led to a highly viscous mass with incomplete polymer dissolution in NMP. However, cellulose acetate butyrate demonstrated greater solubility at high concentrations than other polymers, such as nitrocellulose [14,20] and zein [24], as previously reported. High polymer loading enabled the formation of a robust polymeric matrix to encapsulate the drug and control its release. The amount of cellulose acetate butyrate dissolution in these formulations also aligns with the polymer concentrations used in commercial products like Atridox^®^ [8]. Atridox^®^ contains doxycycline hyclate, using 36.7% poly(DL-lactide) as a polymer to control the drug release [8]. Despite this, it is essential to evaluate the physicochemical properties and efficacy of these formulations as drug carriers for the local delivery of antibacterial agents. Prior to phase transformation induced by water, these in situ gels initially exist in a solution state [8,14].

**Figure 3:**
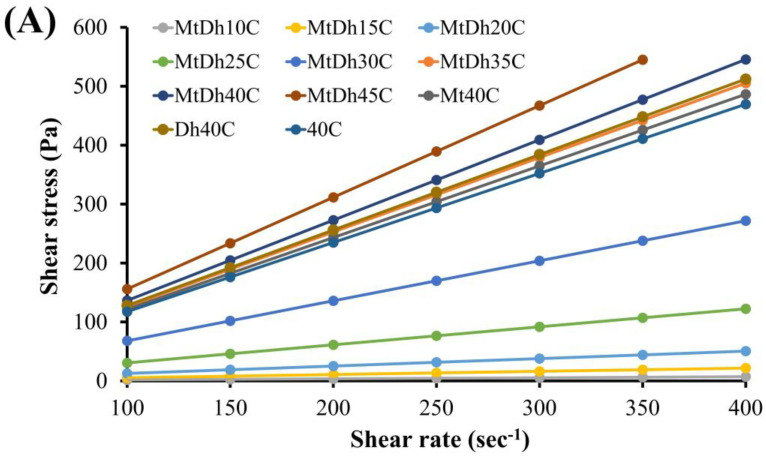

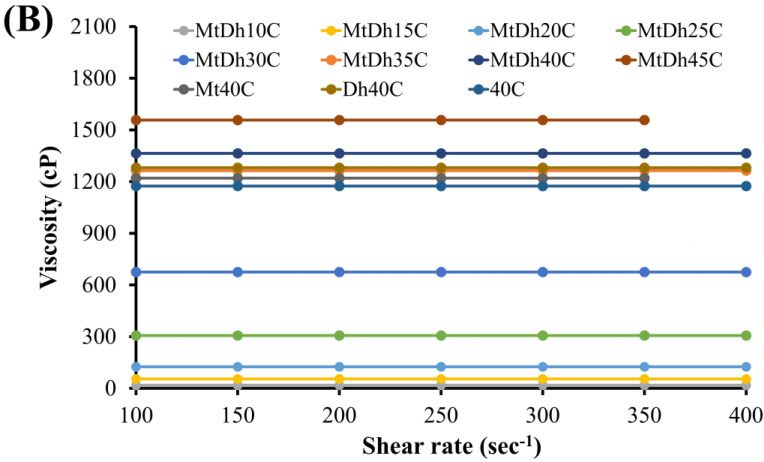
Rheological behavior of metronidazole combined with doxycycline hyclate-dissolved cellulose acetate butyrate-based in situ gel and control formulations for shear stress (**A**) and viscosity (**B**) with shear rate at room temperature.

Apparently, the increasing cellulose acetate butyrate concentration enhanced the viscosity of the formulation, as shown in Figure 3A,B and Table 2. The drug solutions without polymer addition exhibited low viscosity. The incorporation of drug compounds slightly increased the viscosity due to the replacement of the solvent with dissolved drug molecules. The combined drug-loaded 40% polymer solution showed higher viscosity than the drug-free and single drug-loaded polymer-based solutions (*p* < 0.05). The viscosity of 40C was significantly lower than that of Mt40C and Dh40C (*p* < 0.05) (Table 2).

All formulations displayed Newtonian flow, with an increased slope in the shear rate versus shear stress graph observed at higher concentrations of cellulose acetate butyrate (Figure 3A,B). This Newtonian flow behavior and steeper rheological slope have similarly been noted for injectable in situ gel dosage forms containing nitrocellulose [14] and saturated fatty acids [25], as previously mentioned. Achieving a balance between low viscosity, to facilitate injectability into the periodontal pocket, and a sufficiently dense polymer matrix for controlled drug release is essential. This investigation revealed a significant reliance of formulation viscosity on the concentration of cellulose acetate butyrate. Therefore, further evaluations were conducted to assess additional properties of the formulations after transitioning from solution to a gel or matrix-like state. As shown in Table 2, lower injection force values indicated easier needle injection, while higher polymer concentrations increased the solution viscosity and required more injection force. The injectability results corresponded with the viscosity and rheological characteristics of the prepared formulations. The injection force for MtDh40C was significantly greater than that of 40C (*p* < 0.05), indicating that the combined drug loading increased both the viscosity and the resistance to expelling the solution through the needle. The viscosity and injection force required for in situ gels made with small molecules, like borneol [7] and short-chain saturated fatty acids [25], are notably lower compared to those formulated with polymeric materials [18,19,20] as the gel-forming agents. The maximum injection force for all formulations of this investigation remained below 5 N through a 21-gauge needle, suggesting easy injectability and acceptable levels of injection site comfort [14,26]. Consequently, both drug-free and drug-incorporated cellulose acetate butyrate-based in situ gels were considered suitable for injectable drug delivery, ensuring ease of administration and enhanced patient comfort and compliance [26,27].

### 3.2. Contact Angle

Table 2 presents the contact angles used to assess droplet spreadability for all formulations on glass slides and agarose gel surfaces. This study compared the contact angles on a dry surface (glass slide) with those on a wet surface of agarose gel at pH 6.8 to mimic the pH environmental or crevicular fluid in the periodontal pocket. An increase in cellulose acetate butyrate content was observed to raise the contact angle values, which correlated with increased viscosity, as discussed in Section 3.1, thereby reducing the spreadability on glass surfaces. Polymer solutions loaded with a combination of drugs exhibited higher contact angles on glass slides than those without polymers. On agarose gel surfaces, solutions containing 20% cellulose acetate butyrate or more displayed significantly higher contact angles than those on glass slides (*p* < 0.05), as seen in Table 2. For MtDh40C, the contact angle on agarose gel was significantly higher than that of 40C (*p* < 0.05), suggesting that the hydrophilic drugs facilitated phase transition. Solutions of MtN and DhN, as well as NMP droplets, spread almost completely on both surfaces, reflecting good wetting due to their high polarity on hydrophilic surfaces. Greater polymer loading prompted a more pronounced phase inversion, transforming the fluid droplet into a solid-like cellulose acetate butyrate matrix upon contact with the aqueous phase on the agarose surface. This phase transition from a flowable liquid to a condensed droplet reduced spreadability and led to higher contact angles compared to those on dry glass slides [18,19]. Previous studies have shown similar results, such as a study using vancomycin HCl-loaded borneol-based in situ gels, which exhibited reduced spreadability due to phase transformation on wet surfaces [7]. Despite the increased contact angles in these in situ gels, they remained under 60°, indicating adequate wettability on test surfaces [20,25]. Therefore, measuring contact angles, especially on water-enriched surfaces like agarose gel, is an effective technique for illustrating the spreadability of phase-transformed in situ gel formulations influenced by aqueous induction.

### 3.3. Mechanical Properties

To replicate the conditions of full polymeric matrix formation within a periodontal pocket, the prepared solutions were loaded into agarose gel wells for a 72 h period before mechanical testing. Table 2 displays the maximum force of these converted in situ gels. The mechanical integrity of in situ gels after phase inversion is critical for ensuring jaw movement and maintaining structural stability within the periodontal pocket [21]. The drug solutions and NMP without polymer addition could not generate measurable force due to the absence of matrix formation. The formulations containing 40% cellulose acetate butyrate demonstrated similar maximum force values. Nevertheless, drug loading appeared to disrupt the polymeric matrix, resulting in a slight reduction in maximum force. Previous studies have noted similar findings, where the incorporation of antibiotics in borneol- or nitrocellulose-based matrices led to decreased maximum force [7,20]. A high proportion of triacetin in the borneol-based in situ gel formulation also contributed to reduced matrix firmness [7]. Cremophor EL addition as a solubilizing agent substantially decreased the maximum force of the transformed matrix, suggesting that this liquid excipient reduced the sturdiness of the fluconazole-loaded ibuprofen-based in situ gel formulation [28].

An upward trend in the maximum penetration force was observed with increased cellulose acetate butyrate concentration, indicating that the matrix structure strengthened as the polymer solidified more effectively. The maximum penetration force showed an upward trend with increasing concentrations of ibuprofen, indicating enhanced matrix sturdiness due to the intensified solidification of ibuprofen in fluconazole-loaded ibuprofen-based in situ gel [28]. Comparable findings were also reported for thermosensitive gels and hydrogels, where an increase in polymer concentration in in situ gel preparations led to similar effects [29,30]. This outcome also correlated closely with the viscosity and rheological profiles shown in Figure 3A,B. Thus, the mechanical properties of the transformed in situ gel generally increased in relation to cellulose acetate butyrate concentration. A denser polymer matrix can influence solvent exchange rates, matrix formation speed, and drug release behavior, further impacting its functional properties.

### 3.4. Gel Formation

**Figure 4:**
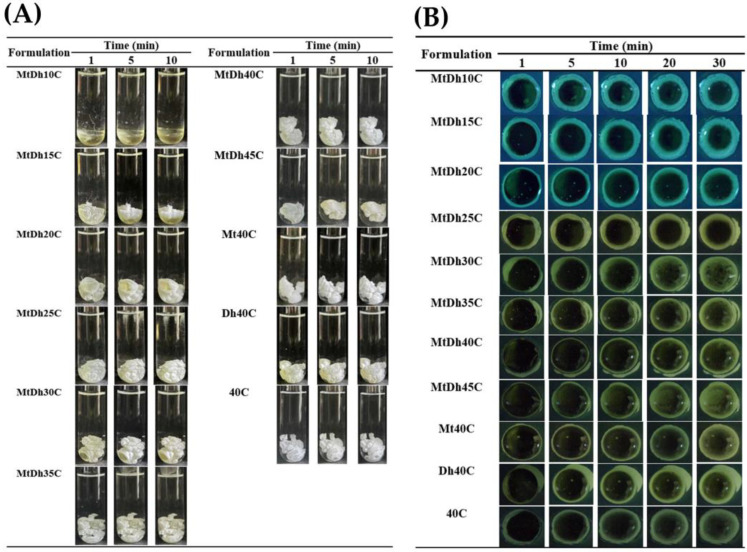
Change in gel formation of metronidazole combined with doxycycline hyclate-dissolved cellulose acetate butyrate-based in situ gel and control formulations in PBS pH 6.8 (**A**) and agarose well (**B**).

The phase inversion process, where prepared formulations transformed from an initial liquid state to a solid-like opaque mass following exposure to PBS pH 6.8 in both glass tubes and agarose gel wells, is illustrated in Figure 4. For formulations lacking cellulose acetate butyrate, including DhN and MtN, no phase separation was observed in either PBS pH 6.8 or agarose gel indicating miscibility with the aqueous phase. However, formulations with low cellulose acetate butyrate content, such as MtDh10C, did not achieve an opaque matrix in PBS pH 6.8 (Figure 4A). This buffer solution, used as a simulated periodontal fluid in several studies [14,18,19,20], was insufficiently effective in promoting a phase transition due to the low polymer concentration in MtDh10C.

In contrast, MtDh15C initially showed a partial transition, forming an opaque gel-like layer around the outer skin of the formulation in the simulated fluid. Over time, this gel layer dissolved, leaving a yellowish liquid primarily settled at the bottom. The rapid phase separation at the outset caused only a thin transformed layer that eventually reverted to a liquid state. The inner parts of the formulation, less exposed to PBS pH 6.8, faced difficulty in solvent exchange, impeding complete phase inversion. This behavior aligns with previous findings on in situ gels with low polymer content, such as moxifloxacin HCl-based gels for periodontal delivery [14] and levofloxacin HCl-salicylic acid systems [20]. A skin-like cloudy mass initially formed around the formulation, progressing to a phase transformation in the interior liquid. Formulations with higher cellulose acetate butyrate content, in contrast, promptly transformed into a stable opaque mass after exposure to simulated periodontal fluid, as shown in Figure 4A. The adequate cellulose acetate butyrate concentration facilitated swift phase inversion, resulting in robust, solid-like matrices.

In the cross-sectional view (Figure 4B), direct contact between the formulation and the agarose rim initiated matrix formation across all formulations. Over time, formulations with low polymer loading, such as MtDh10C and MtDh15C, developed a relatively large opaque matrix ring. The reduced polymer barrier and lower viscosity facilitated easier solvent exchange, resulting in a broad band of phase transition. This feature is characteristic of solvent-exchange-induced in situ gel formulations [14,24].

Upon exposure to the agarose rim, all formulations underwent a rapid phase transformation that gradually progressed inward (Figure 4B). This process led to the formation of an initial gel, followed by a solid matrix, depending on the remaining solvent concentration in each region over time [31,32]. Commercial treatments for periodontitis, like Atridox^®^, use a formulation of 36.7% poly(DL-lactide) (PLA) in 63.3% NMP [33]. Various studies have explored other polymers with antibacterial compounds, typically using polymer concentrations below 35% w/w for periodontitis treatment [14,20,24]. Sufficient polymer content is essential for controlled drug release; however, it should not increase viscosity to the point of hindering injection through a needle. A rapid gel-to-matrix transformation helps minimize burst drug release, making it critical to strike an optimal balance between viscosity and polymer concentration for the sustained drug release of injectable in situ gel formulation.

As the cellulose acetate butyrate content increased in the formulation, the mass became more visibly convex and firm, which corresponds with previous findings on contact angle and gel formation properties. Higher polymer concentrations assist in preserving shape, resembling the behavior observed in shape-memory polymers [34]. Polymer chains exhibit varying degrees of intra-molecular motion, with translational diffusion as the most fundamental (zero order) movement. As the polymer concentration rises, these motions extend beyond simple diffusion to include other forms of intramolecular and eventually cooperative, collective movement [21]. The gel formation experiments highlight the critical influence of cellulose acetate butyrate content concentration on the morphological and phase inversion characteristics of in situ gel formulations. In comparison, using small molecules such as fatty acids, camphor, ibuprofen, and borneol as the matrix-forming agents in in situ gels promotes faster matrix growth than polymeric-based systems [7,25,35]. This difference may be due to the ease of crystal growth in the lower-viscosity environment of small molecule-based in situ gel formulation, whereas the higher viscosity of polymeric-based in situ gel formulations, with their dense matrix barriers, retards solvent exchange and consequently matrix formation over time [20,25].

### 3.5. Microscopic Interfacial Change

**Figure 5:**
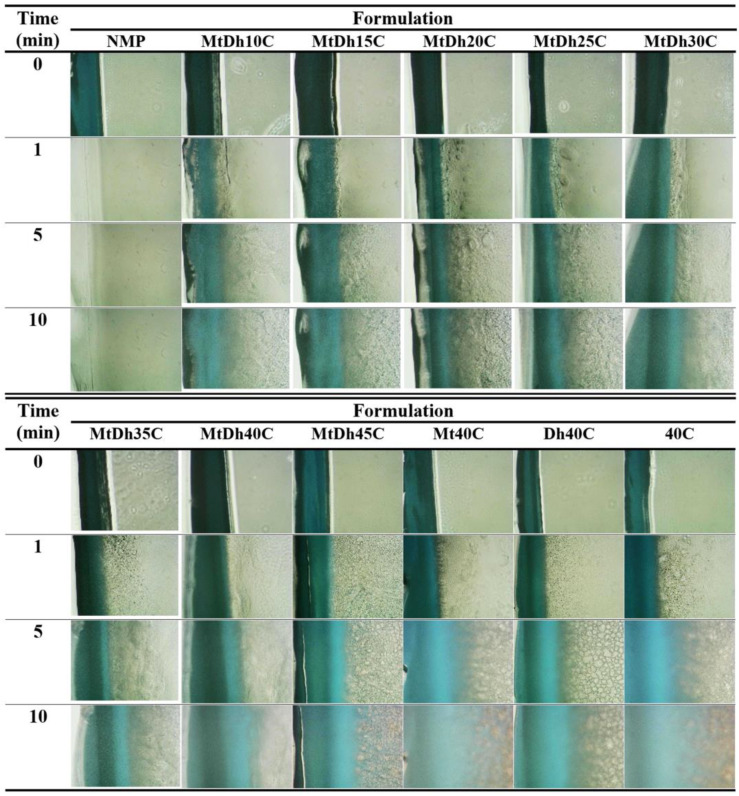
Interface interaction behavior of metronidazole combined with doxycycline hyclate-dissolved cellulose acetate butyrate-based in situ gel and control formulations (right) with agarose gel (left) under inverted microscope (40×).

After the prepared formulations (right) came into contact with the agarose gel (left) at the interface between these two phases, their morphological changes were examined under an inverted microscope, as shown in Figure 5. Upon interface contact with the agarose gel, the formulation facilitated the diffusion of its NMP into the agarose gel while allowing aqueous PBS to diffuse out from agarose into the formulation. All prepared formulations containing cellulose acetate butyrate displayed a progressively larger and rougher mass at the interface, with more expansion towards the formulation side over time. They quickly transitioned into a gel state, forming a dense, streaky cellulose acetate butyrate mass at the interfacial region due to NMP removal. Lower polymer-loaded formulations, such as MtDh10C and MtDh15C, exhibited less rough mass.

This phase separation of the polymer, triggered by its insolubility upon exposure to the aqueous phase of agarose, initiated these microscopic changes [19]. The gelation process of cellulose acetate butyrate through phase separation intensified over time, as depicted in the figure. In contrast, NMP without a polymer-forming matrix showed no significant change. Small molecules like fatty acids, camphor, ibuprofen, and borneol-based in situ gels similarly underwent interfacial phase separation, forming a thicker crystalline mass in this region, which expanded over time in a concentration-dependent manner [7,25,35].

**Figure 6:**
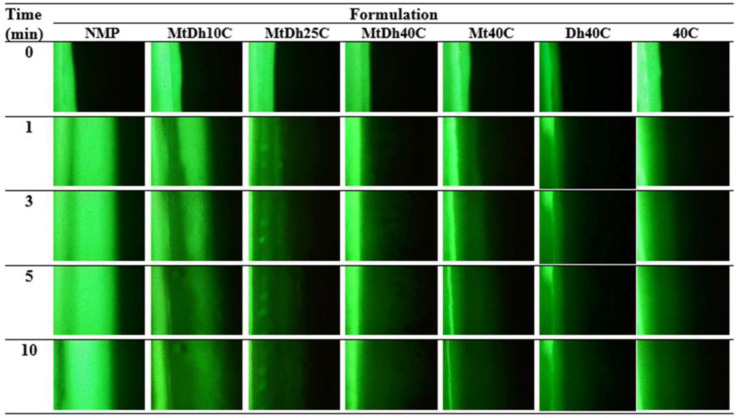
Interface interaction between 0.4 µg/mL of sodium fluorescence-loaded agarose gel (left) against noncolored formulations (right) under inverted fluorescence microscope at magnification of 40×.

**Figure 7:**
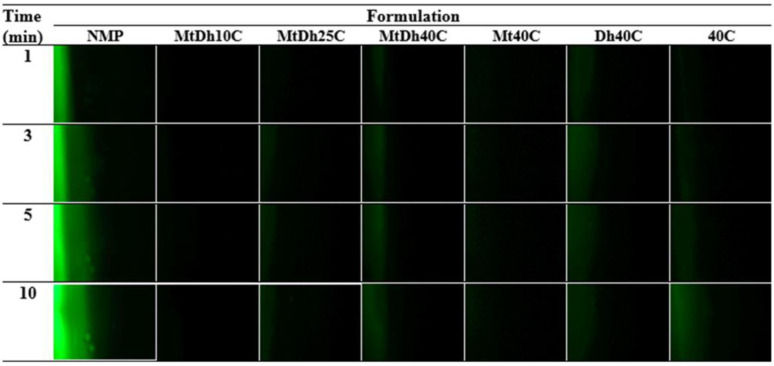
Interface interaction between noncolored agarose gel (left) against 4 µg/mL of sodium fluorescence-loaded formulations (right) under inverted fluorescence microscope at magnification of 40×.

**Figure 8:**
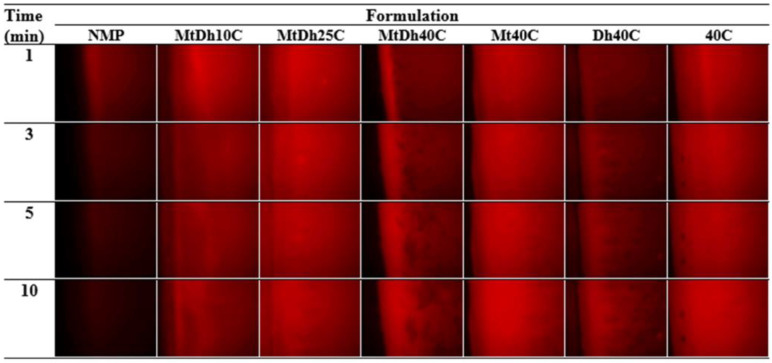
Interface interaction between noncolored agarose gel (left) against 2 µg/mL of Nile red-loaded formulations (right) under inverted fluorescence microscope at magnification of 40×.

**Figure 9:**
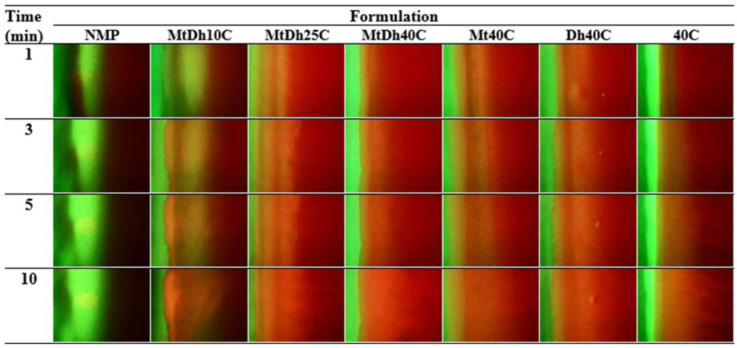
Interface interaction between 0.4 µg/mL of sodium fluorescence-loaded agarose gel (left) against 2 µg/mL of Nile red-loaded formulations (right) under inverted fluorescence microscope at magnification of 40×.

Hydrophilic and hydrophobic fluorescent dyes were employed to clearly and accurately trace the movement exchange between NMP and PBS of the in situ gel formulation and simulated periodontal pocket fluid, respectively. Sodium fluorescein, a dye emitting green in an aqueous environment and quenching in organic solvents, and Nile red, a lipophilic dye emitting red in organic solvents and quenching in water, were used to observe the movement of PBS and NMP at the interface as the formulation solution made contact with the aqueous fluid of agarose gel under an inverted fluorescence microscope [14,36]. The photographs from four different conditions of contact are depicted in Figure 6, Figure 7, Figure 8 and Figure 9.

Figure 6 illustrates the color emission contrast between sodium fluorescein-loaded agarose gel (left) and the non-colored formulations (right). The bright green fluorescence from the agarose gel visibly migrated into the NMP, indicating a rapid transfer. This swift movement was also evident in the MtDh10C formulation, highlighting its limited ability to restrict the diffusion of the aqueous phase from agarose into the formulation. Although MtDh25C allowed some diffusion of the sodium fluorescein along with the aqueous phase from agarose, the lower concentration and quenching in the NMP led to an insufficient green emission. Conversely, formulations containing 40% cellulose acetate butyrate effectively hindered the diffusion of this hydrophilic dye, maintaining most of the green fluorescence within the agarose gel on the left side. This phenomenon is consistent with other polymeric in situ gel delivery systems induced by solvent exchange [19,24]. The gradual transfer of sodium fluorescein, accompanied by an adequate amount of water from the agarose, led to a more intense green color in the formulation. The concentration of matrix-forming agents plays a critical role in controlling solvent and antisolvent migration, which, in turn, influences drug release modulation and antimicrobial activity efficacy [25,35]. An analysis of the impact of the matrix-forming agent on the migration of the solvent and aqueous phase tracking with this technique were also performed for lincomycin HCl-loaded borneol-based in situ gel as previously reported [37]. Therefore, examining the interface boundary and monitoring the movement of sodium fluorescein yields important insights into the water’s mobility and the role of matrix formation on this migration during phase inversion.

In an organic aprotic solvent like NMP, sodium fluorescein does not display fluorescence, resulting in a black background in the images. However, when dissolved in a medium with a high water content, it produces bright green fluorescence. The presence of green fluorescence after contact between noncolored agarose gel (left) and sodium fluorescein-loaded formulations (right) is shown in Figure 7. The bright green fluorescence of sodium fluorescein was clearly present in the agarose gel; thus, this dye quenching in the formulation enabled the dye to emit its green fluorescence when it moved and made contact with water from the agarose gel and enabled it to penetrate into the agarose gel mass. However, the dye may have difficulty diffusing through the cellulose acetate butyrate matrix, or the limited amount of dye that penetrated may not be able to overcome the dilution effects caused by the co-existing NMP, leading to insufficient emission of green fluorescence in the agarose gel matrix. Nonetheless, slight green fluorescence was observed in the MtDh40C, Dh40C, and 40C formulations, suggesting that some of the dye remained in the formulations as it dissolved in the aqueous phase migrating from the agarose gel. This fluorescent probe tracking technique is less complicated than previous research determining the content of different solvent diffusions from polymeric in situ gel and in situ microparticles prepared from bleached shellac into the aqueous medium by using the HPLC method [38]. Solvent diffusion from in situ gels appeared slower than from in situ microparticle systems, as the hydrophobic nature of the essential oil in the external phase of in situ microparticles effectively slowed solvent diffusion. However, interpreting the amount of solvent released at different time intervals would provide insights into the release kinetics. Nonetheless, the application of this hydrophilic fluorescent dye loaded in the formulation was a useful probe for checking the effect of the polymer in the formulation on the retardation of solvent removal and drug release [19,20]. In conclusion, the miscibility between NMP and the aqueous phase enabled sodium fluorescein to diffuse from NMP into the agarose gel, triggering green fluorescence on the left side, while the developing polymer matrix restricted probe diffusion into the gel.

Typically, Nile red, a hydrophobic probe insoluble in the aqueous phase, displays vivid red fluorescence in organic solvents and when incorporated into hydrophobic materials like lipids; however, its fluorescence is quenched in aqueous environments [14,36]. When dissolved in an organic aprotic solvent like NMP, Nile red typically emits fluorescence, whereas in water, the fluorescence is quenched, producing a dark background in photographs [36,37]. Figure 8 illustrates the time-dependent color change between a plain agarose gel (left) and Nile red-loaded in situ gel formulations (right). Notably, Nile red, initially dissolved in pure NMP, showed a gradual decrease in red fluorescence upon contact with the agarose gel. This quenching occurred as water from the agarose gel diffused freely into the NMP, resulting in Nile red losing its fluorescence over time [18]. Observations suggest that Nile red migrated from the MtDh10C and MtDh25C regions into the agarose gel. This migration likely resulted from the transport of Nile red along with NMP through a thin polymer matrix into the agarose gel. However, in formulations with 40% cellulose acetate butyrate, a distinct, intense red fluorescence remained concentrated within the formulation, while the agarose gel displayed a dark background. This implies that the higher polymer content effectively delayed NMP diffusion into the agarose gel. Nevertheless, the inherent color of doxycycline hyclate might slightly interfere with the shade of fluorescence emission.

Additionally, the interaction at the phase interface between the sodium fluorescein-loaded agarose gel (left) and the Nile red-loaded formulations (right) is shown in Figure 9. The rapid miscibility between plain NMP and the aqueous phase of the agarose gel led to fading green fluorescence and an apparent disappearance of red fluorescence, as seen in the first column of Figure 9. The increased polarity of the mixed solvent likely reduced Nile red’s solubility, resulting in quenching and a lack of fluorescent intensity. Meanwhile, sodium fluorescein was also unable to maintain its initial bright green fluorescence when a higher amount of NMP was mixed with the aqueous phase [20]. For MtDh10C, both green and red fluorescence noticeably decreased over time. The significant drop in color intensity for both fluorescent probes in low-polymer-loaded formulations indicates reduced efficacy in preventing the solvent exchange process [19,20].

Formulations containing up to 25% cellulose acetate butyrate maintained both fluorescence colors (Figure 9), suggesting that an adequate concentration of the polymer supported matrix formation upon contact with agarose. As a result, NMP penetration into the agarose was reduced, along with decreased ingress of the aqueous phase into the formulations. Typically, an increasing viscosity of solvent including polymer solution retards the solvent and drug diffusion rates [38,39]. The diffusion rate can be regulated by adjusting the polymer concentration in the solution [39]. The enhanced strength of the cellulose acetate butyrate matrix, as previously noted for the mechanical properties (Table 2), further slowed the solvent exchange process. Consequently, the use of combined fluorescent probes, such as sodium fluorescein with Nile red, is valuable in examining and mapping the dynamic movement of solvent and aqueous phase modulated by polymeric matrix formation in in situ gel systems. The MtDh40C formulation demonstrated several characteristics that make it a promising candidate for an in situ gel dosage form tailored for localized drug delivery to periodontal pockets. Notably, it exhibited Newtonian flow behavior, ease of injectability, rapid and stable matrix formation, and retardation of solvent exchange. These attributes render MtDh40C particularly suitable for further evaluations of drug release and permeation, especially when compared with formulations like MtDh25C.

### 3.6. Drug Content and In Vitro Dual-Drug Release

MtDh40C shows promise as an in situ gel for periodontal drug delivery due to its Newtonian flow, injectability, rapid matrix formation, and reduced solvent exchange, warranting further dual-drug release testing compared to drug-loaded formulations like MtDh25C and control formulations without added polymers (MtN and DhN). The metronidazole or doxycycline hyclate content in these four formulations was analyzed prior to the dual-drug release test. The HPLC analysis confirmed a strong linear relationship between the concentrations of metronidazole and doxycycline hyclate (20–100 µg/mL) and their respective peak area values, with determination coefficients (R^2^) of 0.9983 for metronidazole and 0.9972 for doxycycline hyclate. The regression equations derived were y = 85.84x − 276.2 for metronidazole and y = 39.685x − 175.7 for doxycycline hyclate, establishing the method’s accuracy in quantifying both compounds within the specified range. The limits of detection (LOD) and quantification (LOQ) for metronidazole were 0.0580 µg/mL and 0.1757 µg/mL, respectively; for doxycycline hyclate, these values were 0.1581 µg/mL and 0.4792 µg/mL. The content of metronidazole in MtN, MtDh25C, and MtDh40C was measured at 100.85 ± 2.31%, 98.06 ± 1.72%, and 97.40 ± 0.36%, respectively. The doxycycline hyclate content in DhN, MtDh25C, and MtDh40C was 98.63 ± 1.85%, 101.42 ± 1.61%, and 99.24 ± 1.92%, respectively. Therefore, nearly 100% drug content was achieved through straightforward continuous mixing until clear solutions formed, without the need for heating or complex preparation procedures.

**Figure 10:**
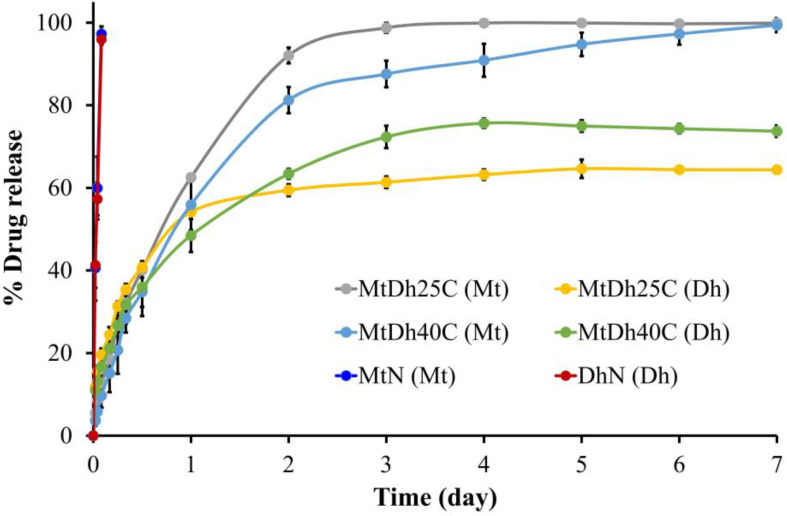
In vitro release profiles of metronidazole and doxycycline hyclate from control, MtDh25C, and MtDh40C formulations in PBS pH 6.8 (n = 3).

Metronidazole and doxycycline hyclate dissolved rapidly in PBS at pH 6.8 from MtN and DhN, respectively, as shown in Figure 10. This rapid dissolution is due to the absence of a polymeric network that could regulate drug release, allowing these drugs to diffuse freely into the release medium. Saliva pH decreases as periodontal disease severity increases [40,41]. Additionally, as salivary pH lowers, the likelihood of missing teeth rises [41]. Under normal oral conditions, saliva maintains a near-neutral pH, approximately 7.06. In patients with chronic periodontitis, however, the average oral pH drops to 6.85, indicating slight acidemia [40]. Therefore, a release test for both control solutions and two selected formulations was conducted in PBS at pH 6.8.

In contrast, the initial burst release of the drugs was reduced, achieving a prolonged release in MtDh25C and MtDh40C over a period of 7 days, as illustrated in Figure 10. The higher cellulose acetate butyrate content in MtDh40C slowed metronidazole release compared to MtDh25C. However, doxycycline hyclate release from MtDh25C after the first day was slower than from MtDh40C. Overall, metronidazole release was faster than the release of doxycycline hyclate. The smaller molecular size of metronidazole, depicted in Figure 1B, facilitated its more efficient diffusion through cellulose acetate butyrate compared to doxycycline hyclate (Figure 1A) into the dissolution medium. Moreover, the incomplete release of doxycycline hyclate (Figure 10) could be attributed to its retention within the polymer matrix. This trend of extended drug release suggests that the cellulose acetate butyrate matrix, following phase inversion, effectively delayed drug release in the in situ gel formulations compared with that of MtN and DhN. Besides the higher viscosity of these two chosen formulations to retard drug diffusion, their phase inversion into the matrix mainly played a key role in extending drug liberation into the release fluid. Upon contact with PBS, this dosage form solidified immediately, consistent with prior gel formation findings in this study, and thus did not interfere with drug analysis in the release medium. NMP, which absorbs UV below 200 nm, did not interfere with the detection of metronidazole at 320 nm or doxycycline hyclate at 347 nm, as analyzed by HPLC.

The incorporation of cellulose acetate butyrate formed a matrix that not only reduced the initial burst release but also provided extended dual-drug release. Its ability to modulate the release of benzydamine HCl independently of drug loading in in situ gels has been recently reported [19]. In addition, cellulose acetate butyrate is a type of cellulose ester in which the hydroxyl groups on the glycoside rings of cellulose are replaced, either partially or fully, with acetyl and butyryl groups. The synthesis process allows for adjustment of the acetyl-to-butyryl substitution ratio, enabling the modification of its properties to suit specific applications. For periodontitis treatment, local drug delivery systems like Atridox^®^ are administered after cleaning the periodontal pocket with an irrigating solution, such as chlorhexidine, to reduce plaque bacteria prior to applying a sustained-release system [42]. Encapsulating metronidazole and doxycycline hyclate in the cellulose acetate butyrate matrix with rapid phase inversion thus prolonged the release of both drugs for a week or more. Additionally, cellulose acetate butyrate is biodegradable, biocompatible, and non-toxic [15,16,17], and it is also more affordable than poly (lactic-co-glycolic) acid, commonly used in commercial products. Therefore, cellulose acetate butyrate is a promising bioplastic polymer for use as a matrix-forming component in in situ gels.

Typically, estimating kinetic parameters and assessing goodness-of-fit through profile fitting provides valuable insights into drug release mechanisms, essential for optimizing drug delivery systems [24]. These parameters, estimated using five mathematical release models, are presented in Table 3. Most drug release profiles fit well with Higuchi’s model, suggesting that drug release was proportional to the square root of time, resulting in a gradual, diffusion-controlled release due to the hydrophobic cellulose acetate butyrate matrix [19,43]. Additionally, the release profile was well matched with the Korsmeyer–Peppas equation; for doxycycline hyclate, the release exponent (n) approached 0.45, indicating a primarily Fickian diffusion mechanism, where drug release was governed more by diffusion than by polymer relaxation [19]. Fickian diffusion is commonly observed in sustained drug delivery systems containing a matrix that entraps and regulates drug release [43]. Higher initial release rates were followed by a slower phase, as drug diffusion encountered a longer path length within the environmental fluid due to saturated concentration [44]. The n value for metronidazole release from MtDh40C indicated anomalous release behavior (n value of 0.7830), aligning with the first-order kinetics, where the release rate depends on the drug concentration remaining in the system [45]. Similarly to doxycycline hyclate, which is effectively modulated in release from bleached shellac- and borneol-based in situ gels [7,38], metronidazole was also controlled in release via high-concentration camphor-incorporated in situ gels [36].

### 3.7. Topography from SEM and XTM

**Figure 11:**
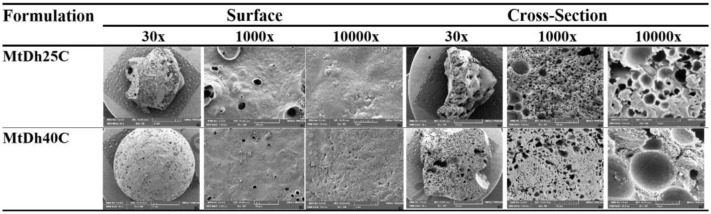
SEM images depicting surface and cross-section view of MtDh25C and MtDh40C after release study at magnifications of 30×, 1000×, and 10,000×.

**Figure 12:**
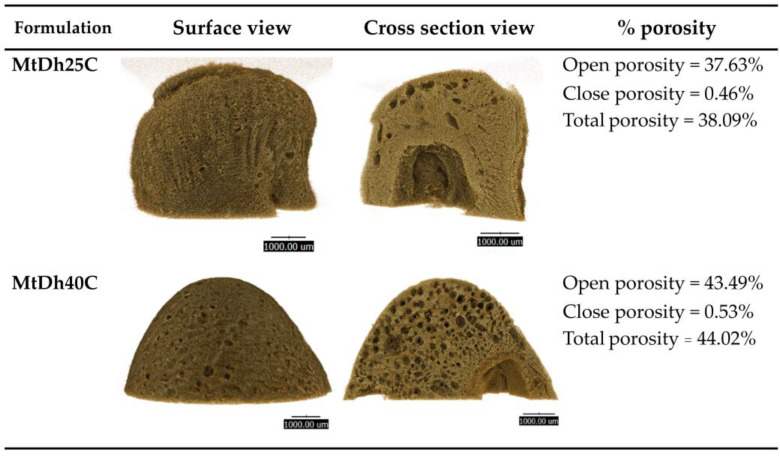
X-ray tomography image and percentage porosity of MtDh25C and MtDh40C formulations after drug release test.

The remnant sample of MtDh25C and MtDh40C following the release test was examined for their topography using two distinct techniques. The matrix of MtDh40C showed a more consistent structure compared to MtDh25C, particularly noticeable in the surface morphology when examined via SEM (Figure 11) and XTM (Figure 12). SEM comparisons of the surface and cross-sectional views of both matrices revealed significant differences in morphological structure. Surface images depicted a relatively smoother texture with fewer open pores, whereas cross-sectional images showed a rugged surface with a scaffold-like network of pores in the cellulose acetate butyrate matrix. This structure emerged as the matrix formed through solvent exchange from a polymer solution. The SEM analysis of MtDh25C indicated a higher number of and larger pores on its surface than MtDh40C, suggesting that a higher polymer concentration fosters a more continuous matrix surface. However, scaffold-like structures appeared in the cross-sectional views of both matrices (Figure 11 and Figure 12).

This scaffold formation likely resulted from the outward diffusion of NMP and the phase separation of the polymer upon exposure to an aqueous environment. Such a scaffold morphology induced by solvent exchange in in situ gels has been documented in polymer-based drug delivery systems [24,38], contrasting with the layered slab matrix often formed by small-molecule crystallization in in situ gels [25,28]. This solvent exchange process facilitated the formation of a microporous polymeric structure. Consequently, the encapsulated drug dissolved and gradually released through the polymeric network and porous structure, further enhancing its porosity over time. Similar effects have been observed in the development of PLGA-based [46] rosin-based [47], and other-polymer-based in situ gels [14,24,39]. Although MtDh40C displayed higher porosity, as shown by XTM, it exhibited a more homogeneous, continuous surface in SEM, acting as a principal barrier to drug release. Additionally, MtDh25C showed more pronounced surface erosion in both SEM and XTM, which could facilitate drug release from the surface. Therefore, the observed morphology of the remnants aligns well with the previously described drug release behavior. SEM provided detailed localized structural information, whereas XTM delivered a comprehensive view of the entire residual matrix. Furthermore, the highly concentrated polymeric in situ gel, like MtDh40C, developed a more robust and consistent matrix structure with a uniformly porous mass.

### 3.8. Drug Permeation

Pathogen invasion into superficial gingival tissues has been documented [48]. Bacteria associated with gingivitis can penetrate deeper layers of tissue and the surrounding periodontium [49,50]. Consequently, investigating antibiotic drug permeation is important to assess its potential to eliminate such pathogens and related microbes. To simulate the pH of crevicular fluid- or saliva-covered superficial buccal tissue, phosphate-buffered saline (PBS) at pH 6.8 was used as the receptor compartment for buccal membrane permeation. Due to the hardness and challenging preparation of gingival tissue as a barrier membrane [51], porcine buccal membrane was selected for ease of preparation and suitability for use in Franz diffusion cells [20]. This study therefore employed porcine buccal mucosa as the membrane to evaluate dual-drug permeation from two in situ gels, focusing on permeation into the buccal membrane. Additionally, the dual-drug accumulation within the porcine mucosa and retention in the in situ gels was analyzed.

**Figure 13:**
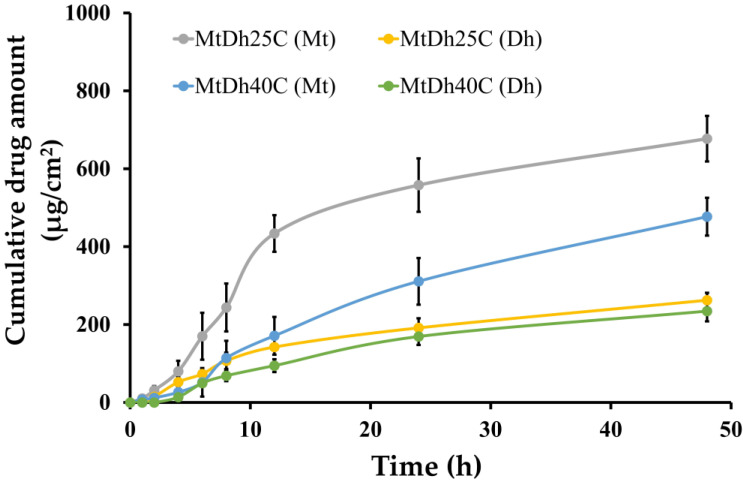
Permeation of metronidazole and doxycycline hyclate from MtDh25C and MtDh40C formulations through porcine buccal membrane (n = 3).

The permeation of metronidazole and doxycycline hyclate from the MtDh25C and MtDh40C formulations through the porcine buccal membrane is presented in Figure 13, with the corresponding flux and lag times shown in Table 4. Comparing the penetration rates of both drugs, it was observed that drug permeation from MtDh40C was slower than from MtDh25C. Notably, doxycycline hyclate permeated at a slower rate than metronidazole from both in situ gel formulations. These findings are consistent with previous drug release studies, indicating that higher cellulose acetate butyrate loading results in slower drug diffusion. This suggests that formulations with higher polymer content exhibit reduced permeation rates due to the denser cellulose acetate butyrate matrix, similar to the effect observed when using nitrocellulose as the matrix-forming agent in in situ gels [20].

Metronidazole exhibits poor water solubility and hydrophobic characteristics, with a solubility of 10.5 mg/mL in water at 25 °C and a low log*p* value of −0.18, classifying it as a hydrophilic drug [52]. Doxycycline hyclate, on the other hand, is highly water-soluble (>50 mg/mL) and is also considered hydrophilic, with a log*p* of −0.25 [53]. The relatively higher hydrophobicity of metronidazole led to increased permeation into the test tissue. The flux values presented in Table 4 confirm this permeability pattern. Both drugs demonstrated lag times of approximately 1.5 h, except for doxycycline hyclate from the MtDh25C formulation, which exhibited a shorter lag time of 0.57 ± 1.02 h, likely due to its high solubility and ease of diffusion from this formulation [20,54].

Table 5 shows the accumulation of each drug in the donor chamber, buccal tissue, and receptor chamber. Metronidazole permeated more effectively, with a greater presence in the receptor chamber PBS than in the buccal tissue and donor compartment. The higher accumulation of metronidazole from MtDh25C in PBS and buccal tissue, compared to MtDh40C, reflects the easier permeation from the formulation with lower polymer loading. The greater retention of metronidazole in the donor compartment of MtDh40C supports controlled release into crevicular fluid, which could inhibit pathogens in the periodontal pocket area [20]. Conversely, doxycycline hyclate predominantly accumulated in the tissue, followed by the receptor and donor chambers. The higher accumulation of doxycycline hyclate from MtDh25C in PBS and tissue, compared to MtDh40C, similarly indicates easier permeation from the less concentrated cellulose acetate butyrate matrix. The pronounced tissue presence of doxycycline hyclate is advantageous for targeting pathogens within the superficial gingiva or pathogenic biofilms [49,50]. The gradual release into the receptor chamber remained dependent on polymer concentration, and NMP’s effectiveness as a permeation enhancer through the tissue, likely via a cotransport mechanism [54,55], suggests that its use could aid in eradicating invading periodontal pathogens. The antimicrobial efficacy of these in situ gels against periodontitis-related microbes is discussed further in the following section.

### 3.9. Antimicrobial Activities

**Figure 14:**
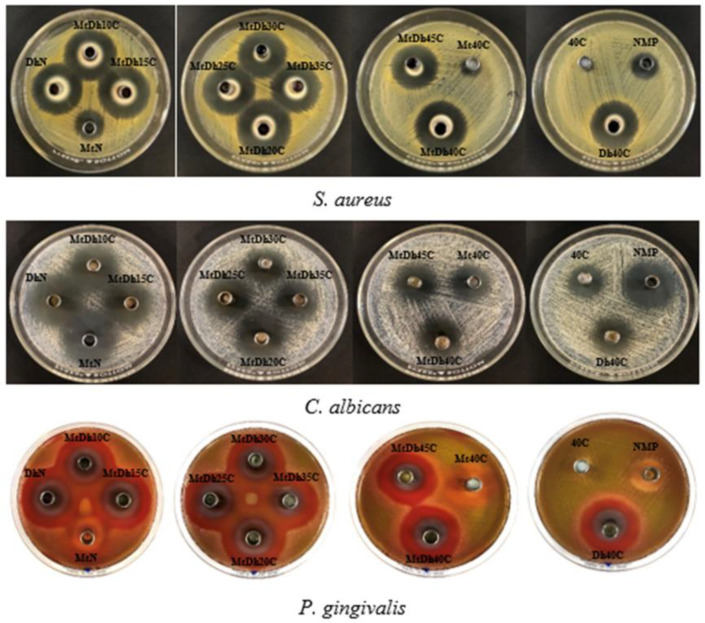
Photographs of the inhibition zone of metronidazole combined with doxycycline hyclate-dissolved cellulose acetate butyrate-based in situ gel and control formulations against *S. aureus*, *C. albicans*, and *P. gingivalis*.

The results of the antimicrobial activities of all test samples and control formulations against various microorganisms, including *S. aureus* ATCC 6538, *C. albicans* 10231, and *P. gingivalis* ATCC 33277, are presented in Table 6 and Figure 14. Both *S. aureus* and *P. gingivalis* are well-known pathogens associated with dental plaque and periodontal diseases, with *P. gingivalis* being a significant focus in antimicrobial testing due to its role as a primary pathogen among anaerobic *Gram*-negative bacteria linked to periodontitis [1,2,3]. Likewise, *S. aureus* and *C. albicans* have been occasionally detected in the periodontal pockets of patients with aggressive periodontitis [38,56].

In comparative testing, the drug-free control formulations, such as 40C, demonstrated the smallest inhibition zone diameter against *S. aureus* and no inhibition zone for *P. gingivalis*. This minimal antibacterial effect likely resulted from limited diffusion of NMP into the agar medium from the cellulose acetate butyrate matrix. NMP, known for its mild antimicrobial properties [53,57], displayed some degree of inhibitory effect on all three tested microbes, as shown in Table 6 and Figure 14. In contrast, doxycycline hyclate solution (DhN) demonstrated superior bacterial inhibition compared to metronidazole solution (MtN), likely due to doxycycline’s broader antibacterial spectrum, which enhances its inhibitory efficacy [7,8].

The metronidazole–doxycycline hyclate-loaded, low-content cellulose acetate butyrate-based in situ gels, including MtDh10C and MtDh15C, demonstrated larger inhibition zone diameters against two bacterial strains compared to the single drug-loaded solutions (DhN and MtN). This result suggests a synergistic effect between the two drugs in inhibiting bacterial growth, as previously reported by Charoensuksai et al. [12]. This finding emphasizes the benefits of incorporating metronidazole-doxycycline hyclate in an in situ gel system, showcasing the synergistic advantages of this drug combination. However, as the polymer concentration increased, a trend of decreasing inhibition zone diameter was observed. This effect can be attributed to the greater restriction on dual-drug diffusion, as higher polymer concentrations form a denser matrix that slows drug release into agar media inoculated with bacteria, thereby reducing the inhibition zone [19,35].

A notable reduction in the inhibition zone was observed for Mt40C compared to Dh40C, suggesting that the more hydrophilic doxycycline hyclate, with higher water solubility, diffused more readily from the polymer matrix into the agar medium to inhibit bacterial growth. A similar trend was observed for antifungal activity against *C. albicans*, although in this case, NMP diffusion played the primary role in inhibiting fungal growth, as shown in Table 6 and Figure 14. Both metronidazole and doxycycline hyclate exhibited minimal antifungal activity, with NMP’s dominant antifungal action previously documented [57]. This property highlights NMP as a valuable solvent for the designed dosage forms, enhancing antimicrobial efficacy.

**Figure 15:**
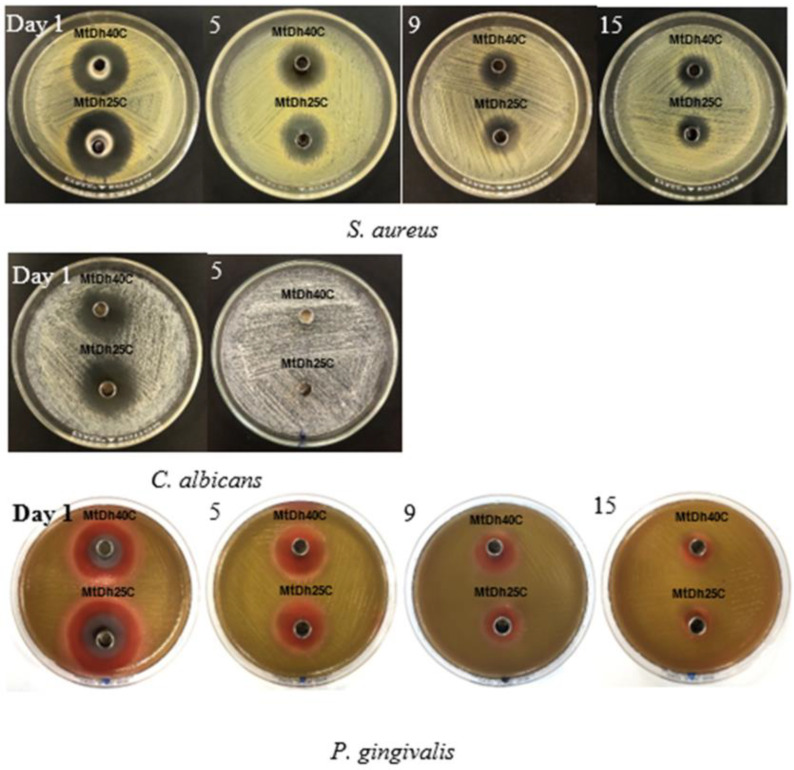
Photographs of the inhibition zone of in situ gel formulations (MtDh25C and MtDh40C) for days 1, 5, 9, and 15 against *S. aureus*, *C. albicans*, and *P. gingivalis*.

The inhibition diameter against *C. albicans* 10231 for MtDh25C and MtDh40C was observed only on the first day after incubation, as shown in Table 7 and Figure 15, with the results consistent with those of the previous test. The formulation with higher cellulose acetate butyrate loading, MtDh40C, displayed a smaller inhibitory zone diameter. No new inhibition zone was observed after transferring the cup containing the transformed matrix of the in situ gel to fresh inoculated agar. This suggests rapid, complete diffusion of the small-molecule NMP from the formulation on the first day. The reduction in NMP, known for its mild antifungal properties [53,57], led to the absence of a clear inhibition zone upon subsequent transfer against *C. albicans*. Nevertheless, this quick initial release of NMP could potentially enhance antifungal activity at the target site. NMP is commonly used in numerous commercial pharmaceutical products, and it exhibits low acute toxicity when administered orally, dermally, or by inhalation [58,59,60]. Atridox^®^ is a commercially available in situ gel formulation comprising doxycycline hyclate-incorporated D,L-lactic acid (PLA) and dissolved in NMP.

Efficient retention of antibacterial activity was achieved for MtDh25C and MtDh40C (Table 7, Figure 15). This experimental design aimed to assess the ability of the transformed polymeric matrix in in situ gels to sustain drug release, thereby extending their bioactivity. Initial observations of the inhibition zone diameter indicated that drug diffusion was higher from MtDh25C, resulting in a larger inhibition zone compared to MtDh40C. A gradual reduction in the inhibition zone was noted over time with each transfer into fresh inoculated agar media. Nevertheless, antibacterial activity was retained up to day 15, highlighting the sustained antibacterial capability of these two in situ gels due to their prolonged dual-drug release properties, as previously reported.

Interestingly, at later observation points, MtDh40C exhibited a larger inhibition zone than MtDh25C against *P. gingivalis* 33277. Additionally, MtDh40C showed a greater inhibition zone than MtDh25C against *S. aureus* 6538 on day 5. These findings suggest effective dual-drug entrapment within MtDh40C and a slower drug diffusion rate into the inoculated media. The denser matrix in MtDh40C, formed by solvent exchange with the aqueous phase from the agar media, effectively modulated dual-drug release, inhibiting bacterial growth [24,25]. Similar results regarding the role of polymer content on drug release and antimicrobial activity were previously observed in in situ gels using zein [24] and nitrocellulose [14] as matrix-forming agents, where higher polymer content increased tortuosity and reduced porosity [61]. This result aligns with their higher contact angle, as shown in Table 2, due to increased viscosity and phase inversion. Atridox^®^ is a commercially available in situ gel formulation that contains PLA and NMP, loaded with doxycycline hyclate, designed for periodontitis treatment by sustaining drug release over one to two weeks [62]. Similarly, the developed cellulose acetate butyrate-based in situ gels loaded with metronidazole and doxycycline hyclate demonstrated sustained drug release. Additionally, their synergistic antibacterial activities are expected to enhance efficacy in periodontitis therapy. Therefore, combined metronidazole and doxycycline hyclate-loaded in situ gels with cellulose acetate butyrate should be formulated to address the treatment needs of periodontitis. NMP can dissolve the lipid components of bacterial cell envelopes, disrupting bacterial nutrient transport. This property makes NMP suitable for use in in situ gel delivery systems for antimicrobial agents, as it can enhance their effectiveness against infectious diseases. Furthermore, NMP’s high bacterial cell membrane permeability and role as a penetration enhancer [58,63] can amplify the antimicrobial effects of antibacterial drugs. The viability assay for Schwann cells and fibroblasts demonstrated that cellulose acetate butyrate nanofibers were non-toxic, highlighting their potential as scaffolds in tissue engineering applications [64,65]. The MtDh40C formulation, in particular, possesses as an injectable dosage form for targeting periodontopathogens, especially *Gram*-negative anaerobes like *Porphyromonas gingivalis*, in periodontitis treatment. Its controlled drug release profile provides effective, sustained synergistic antibacterial activity. However, further investigation through in vitro cytotoxicity testing and clinical trials is necessary to validate its potential for biomedical applications [66].

## 4. Conclusions

In conclusion, this study successfully developed solvent-exchange-induced cellulose acetate butyrate-based in situ gels for the treatment of periodontitis, incorporating combined metronidazole and doxycycline hyclate as active agents. The formulation demonstrated favorable physicochemical properties, including ease of injectability, efficient gel formation, and sustained drug release. As the polymer content increased, the gels exhibited improved matrix formation, leading to a larger contact angle on agarose gel surfaces, while still preserving excellent spreading properties. The in situ gels exhibited controlled drug delivery with minimal initial burst release, achieving a prolonged release profile over 7 days, which was predominantly governed by a diffusion-controlled mechanism. In the dual-drug permeation test using Franz’s diffusion cell, metronidazole and doxycycline hyclate successfully permeated through porcine buccal tissue from the in situ gel, potentially allowing them to target invading bacterial pathogens in superficial tissues. The formulations also showed promising antimicrobial activity against *S. aureus*, *P. gingivalis*, and *C. albicans*, with higher polymer concentrations enhancing antibacterial efficacy through delayed drug diffusion and prolonged retention. Additionally, fluorescence emission studies using sodium fluorescein and Nile red revealed the ability of the cellulose acetate butyrate matrix to slow solvent exchange. These findings indicate that a combination of metronidazole and doxycycline hyclate in a 40% cellulose acetate butyrate-based in situ gel could offer an effective and sustained therapeutic approach for targeting periodontal pathogens, potentially improving clinical efficacy in the treatment of periodontitis. Alternative solvents, including triacetin and glycofurol, will be explored further for their potential to dissolve cellulose acetate butyrate and facilitate the preparation of solvent-exchange-induced in situ gels.

## Figures and Tables

**Table 1:** Compositions of metronidazole combined with doxycycline hyclate-dissolved cellulose acetate butyrate-based in situ gels and control formulations.

Formulation	Amount (% w/w)			
	Metronidazole	Doxycycline Hyclate	Cellulose Acetate Butyrate	NMP
MtN	2			98
DhN		2		98
MtDh10C	2	2	10	86
MtDh15C	2	2	15	81
MtDh20C	2	2	20	76
MtDh25C	2	2	25	71
MtDh30C	2	2	30	66
MtDh35C	2	2	35	61
MtDh40C	2	2	40	56
MtDh45C	2	2	45	51
Mt40C	2		40	58
Dh40C		2	40	58
40C			40	60
NMP				100

**Table 2:** Physical properties of metronidazole combined with doxycycline hyclate-dissolved cellulose acetate butyrate-based in situ gel and control formulations (n = 3).

Formulation	Viscosity (cP)	Injectability (N)	Contact Angle (°)		Hardness (N)
			Glass Slide	Agarose Gel	
MtDh10C	17.90 ± 0.15	0.105 ± 0.011	14.59 ± 0.65	12.62 ± 0.48	1.001 ± 0.452
MtDh15C	54.19 ± 1.32	0.125 ± 0.025	16.72 ± 0.93	19.24 ± 0.37	1.919 ± 0.161
MtDh20C	126.67 ± 2.59	0.130 ± 0.017	17.07 ± 0.84 ^e^	34.10 ± 1.03 ^e^	4.808 ± 0.081
MtDh25C	291.68 ± 19.47	0.127 ± 0.018	20.33 ± 0.88 ^f^	40.52 ± 1.94 ^f^	4.243 ± 1.446
MtDh30C	674.75 ± 17.18	0.133 ± 0.015	23.62 ± 1.03 ^g^	44.51 ± 1.83 ^g^	5.075 ± 0.806
MtDh35C	1291.63 ± 49.63	0.140 ± 0.014	25.31 ± 1.08 ^h^	49.24 ± 2.26 ^h^	4.886 ± 0.691
MtDh40C	1402.41 ± 36.78 ^a^	0.136 ± 0.013 ^d^	28.82 ± 1.90 ^i^	53.51 ± 2.27 ^i,n^	4.849 ± 2.303
MtDh45C	1506.61 ± 88.90	0.146 ± 0.012	29.69 ± 2.00 ^j^	56.39 ± 2.56 ^j^	6.746 ± 0.603
Mt40C	1216.50 ± 20.77 ^a, b^	0.139 ± 0.018	26.82 ± 1.40 ^k^	51.88 ± 2.04 ^k^	4.413 ± 0.811
Dh40C	1280.54 ± 70.04 ^a, c^	0.135 ± 0.029	27.32 ± 1.30 ^l^	53.11 ± 1.36 ^l^	4.400 ± 1.322
40C	1174.04 ± 28.29 ^a, b, c^	0.129 ± 0.011 ^d^	25.97 ± 0.90 ^m^	49.85 ± 1.83 ^m, n^	4.417 ± 1.193
MtN	4.28 ± 0.25	0.062 ± 0.005	3.92 ± 0.33	3.82 ± 0.22	ND
DhN	6.11 ± 0.41	0.074 ± 0.004	4.51 ± 0.35	4.32 ± 0.16	ND
NMP	3.85 ± 0.24	0.0425 ± 0.001	2.36 ± 0.03	1.72 ± 0.44	ND

The superscripts a–d and n in columns and superscripts e–m between columns represent significant differences (*p* < 0.05).

**Table 3:** Estimated parameters of the release of metronidazole and doxycycline hyclate from MtDh25C and MtDh40C formulations in PBS (pH 6.8).

Model	Parameters	MtDh25C (Mt)	MtDh25C (Dh)	MtDh40C (Mt)	MtDh40C (Dh)
Zero-order	k_0_	4.359	5.192	3.627	4.541
	R^2^	0.9011	0.1954	0.9638	0.2518
	AIC	11.2195	13.1218	11.9001	15.3376
	MSC	4.4337	3.8369	4.1196	3.2150
First-order	k_1_	0.052	0.065	0.042	0.055
	R^2^	0.9399	0.3993	0.9763	0.4109
	AIC	22.9530	35.0858	24.2381	18.7408
	MSC	2.4781	0.1763	2.0633	32.7024
Higuchi’s	k_H_	10.417	12.904	8.548	11.256
	R^2^	0.9546	0.9511	0.9090	0.9493
	AIC	21.2718	20.0414	27.0685	19.2631
	MSC	2.7583	2.6837	1.8629	2.2047
Korsmeyer–Peppas	k_KP_	7.985	15.027	5.381	12.945
	R^2^	0.9939	0.9889	0.9917	0.9794
	n	0.665	0.402	0.7830	0.4000
	AIC	11.2195	13.1218	11.9001	15.3376
	MSC	4.4337	3.8369	4.1196	3.2150

**Table 4:** Flux and lag time of metronidazole and doxycycline hyclate of MtDh25C and MtDh40C permeated through porcine buccal membrane.

Drug	Formulation	Flux (µg/cm^2^/h)	Lag Time (h)
Metronidazole	MtDh25C	36.72 ± 4.55	1.58 ± 0.44
	MtDh40C	16.98 ± 4.09	1.42 ± 0.03
Doxycycline hyclate	MtDh25C	13.17 ± 0.19	0.57 ± 1.02
	MtDh40C	9.45 ± 1.29	1.56 ± 0.5

**Table 5:** Metronidazole and doxycycline hyclate remaining from donor chamber, buccal membrane, and receptor chamber of MtDh25C and MtDh40C formulations after permeation test.

Metronidazole content				
Compartment	MtDh25C		MtDh40C	
	(%)	(µg)	(%)	(µg)
Donor chamber	6.30 ± 0.73	126.04 ± 14.69	18.14 ± 0.55	435.82 ± 102.8
Buccal tissue	35.35 ± 6.37	707.05 ± 127.47	32.84 ± 0.69	612.5 ± 26.09
Receptor chamber	56.91 ± 5.12	1138.24 ± 102.35	44.65 ± 1.74	834.71 ± 84.91
**Doxycycline hyclate content**				
**Compartment**	**MtDh25C**		**MtDh40C**	
	**(%)**	**(µg)**	**(%)**	**(µg)**
Donor chamber	8.74 ± 0.33	174.78 ± 6.68	12.17 ± 0.13	227.07 ± 13.57
Buccal tissue	55.88 ± 1.58	1117.65 ± 31.55	51.71 ± 4.22	963.07 ± 54.48
Receptor chamber	22.97 ± 1.67	459.34 ± 33.33	21.96 ± 1.96	410.10 ± 45.72

**Table 6:** Antimicrobial activity of metronidazole combined with doxycycline hyclate-dissolved cellulose acetate butyrate-based in situ gels and control formulations against three microbes (n = 3).

Formulations	Inhibition Zone Diameter (mm ± S.D.)		
	*S*. *aureus* 6538	*C. albicans* 10231	*P. gingivalis* 33277
MtN	16.3 ± 0.6	32.0 ± 1.0	23.3 ± 1.2
DhN	31.3 ± 0.6	30.7 ± 1.5	38.3 ± 0.6
MtDh10C	32.7 ± 2.1	29.3 ± 0.6	40.3 ± 0.6
MtDh15C	31.7 ± 1.5	28.3 ± 0.6	39.0 ± 1.0
MtDh20C	29.0 ± 0.0	28.0 ± 1.0	37.3 ± 0.6
MtDh25C	28.3 ± 0.6	23.0 ± 2.0	36.3 ± 0.6
MtDh30C	29.3 ± 1.2	20.3 ± 0.6	36.0 ± 1.0
MtDh35C	27.3 ± 1.2	19.7 ± 0.6	35.3 ± 0.6
MtDh40C	26.7 ± 1.5	19.7 ± 1.2	33.7 ± 0.6
MtDh45C	26.0 ± 1.0	21.7 ± 1.2	32.7 ± 1.2
Mt40C	10.3 ± 0.6	20.0 ± 1.7	15.0 ± 1.0
Dh40C	26.7 ± 0.6	19.3 ± 1.5	34.3 ± 0.6
40C	3.3 ± 0.0	20.0 ± 1.0	-
NMP	15.0 ± 1.0	26.7 ± 1.5	18.7 ± 0.6

(-) = no inhibition zone.

**Table 7:** Antimicrobial activity of in situ gel formulations (MtDh25C and MtDh40C) for days 1, 5, 9, and 15 against *S. aureus*, *C. albicans*, and *P. gingivalis* (n = 3).

Formulations	Day	Inhibition Zone Diameter (mm ± S.D.)		
		*S. aureus* 6538	*C. albicans* 10231	*P. gingivalis* 33277
MtDh25C	1	29.3 ± 0.6	25.0 ± 1.0	34.3 ± 1.2
	5	18.7 ± 0.6	-	23.7 ± 0.6
	9	12.7 ± 1.5	-	15.0 ± 2.0
	15	10.3 ± 0.6	-	12.0 ± 0.0
MtDh40C	1	26.7 ± 0.6	19.7 ± 0.6	30.7 ± 1.5
	5	20.3 ± 0.6	-	24.7 ± 1.5
	9	12.0 ± 1.0	-	19.0 ± 2.0
	15	7.7 ± 0.7	-	15.3 ± 1.5

(-) = no inhibition zone.

## Data Availability

The data are contained within the article.
